# Study protocol: ICONS: Identifying continence options after stroke: A randomised trial

**DOI:** 10.1186/1745-6215-12-131

**Published:** 2011-05-20

**Authors:** Lois H Thomas, Caroline L Watkins, Beverley French, Christopher Sutton, Denise Forshaw, Francine Cheater, Brenda Roe, Michael J Leathley, Christopher Burton, Elaine McColl, Jo Booth

**Affiliations:** 1 School of Health, University of Central Lancashire, Preston, PR1 2HE, UK; 2 Institute for Applied Health Research, Glasgow Caledonian University, Cowcaddens Road, Glasgow, G4 0BA, UK; 3 Evidence-Based Practice Research Centre, Edge Hill University, St Helens Road, Ormskirk, L39 4QP, UK; 4 Centre for Health-Related Research, Bangor University, Gwynedd, LL57 2EF, UK; 5 Medical School, Newcastle University, Newcastle upon Tyne, NE2 4HH, UK; 6 School of Health, Glasgow Caledonian University, Cowcaddens Road, Glasgow, G4 0BA, UK

## Abstract

**Background:**

Urinary incontinence following acute stroke is common, affecting between 40%-60% of people in hospital after a stroke. Despite the availability of clinical guidelines for urinary incontinence and urinary incontinence after stroke, national audit data suggest incontinence is often poorly managed. Conservative interventions (e.g. bladder training, pelvic floor muscle training and prompted voiding) have been shown to have some effect with participants in Cochrane systematic reviews, but have not had their effectiveness demonstrated with stroke patients.

**Methods/Design:**

A cluster randomised controlled pilot trial designed to assess the feasibility of a full-scale cluster randomised trial and to provide preliminary evidence of the effectiveness and cost-effectiveness of a systematic voiding programme for the management of continence after stroke. Stroke services will be randomised to receive the systematic voiding programme, the systematic voiding programme plus supported implementation, or usual care. The trial aims to recruit at least 780 participants in 12 stroke services (4 per arm). The primary outcome is presence/absence of incontinence at six weeks post-stroke. Secondary outcomes include frequency and severity of incontinence, quality of life and cost-utility. Outcomes will be measured at six weeks, three months and (for participants recruited in the first three months) twelve months after stroke. Process data will include rates of recruitment and retention and fidelity of intervention delivery. An integrated qualitative evaluation will be conducted in order to describe implementation and assist in explaining the potential mediators and modifiers of the process.

**Trial Registration:**

ISRCTN: ISRCTN08609907

## Background

Urinary incontinence following acute stroke is common, affecting between 40%-60% of people in hospital after a stroke; 25% at hospital discharge and 15% after one year [1]. In longer term stroke survivors, the prevalence has been reported as 17% [2]. Urge incontinence is the most common type after stroke [3], but the cerebral lesion may also lead to practical difficulties with bladder control caused by, for example, motor impairment, depression and aphasia [4] (termed functional incontinence). Despite the availability of clinical guidelines for the management of urinary incontinence in general [5] and urinary incontinence after stroke [6], national audit data [7] suggest incontinence is often poorly managed.

Our systematic review [8] found no rigorously conducted studies evaluating interventions designed to manage urinary incontinence after stroke in secondary care. The intervention in our programme will focus on conservative strategies shown to have some effect with participants in studies included in Cochrane systematic reviews [9-13], but which have not had their effectiveness demonstrated with stroke patients. These strategies are a combined package of bladder training and (where possible) pelvic floor muscle training, and prompted voiding.

Available conservative interventions for urinary incontinence include bladder training, timed voiding, prompted voiding, habit retraining and pelvic floor muscle training. Bladder training is generally used for urge incontinence and aims to increase the time interval between voids so continence is regained. It involves patient education, scheduled voiding, and positive reinforcement, but can also include self-monitoring and urge suppression techniques. Prompted voiding and timed voiding have mainly been used with people who have cognitive deficits. They are based on a system of scheduled voids, with prompted voiding including reminders and reinforcement for self-initiation of toileting. To date, trials of prompted voiding have mainly taken place in United States nursing homes; however there is no a priori reason why this approach should not be introduced into the care of stroke patients in secondary care in the UK.

Pelvic floor muscle training may also be effective in assisting the individual to manage urge, stress or mixed incontinence [10], and has been shown to be effective as a combined intervention with bladder training [14,15].

The effectiveness of conservative interventions has been systematically reviewed in adults. In the review of bladder training [11], trials tended to favour bladder training and there was no evidence of adverse effects. The review of prompted voiding [9] found evidence of increased self-initiated voiding and decreased incontinent episodes in the short-term.

Despite a growing evidence-base, existing evidence has not been widely implemented in clinical practice, even by stroke specialist teams working on recognised stroke units [16]. This lack of implementation in stroke clinical practice is in keeping with a recent and growing recognition that the implementation of research in practice is influenced not only by individual clinicians but also by the organisational context in which they operate [17-22]. Organisational context has been defined as "the environment or setting in which the proposed change is to be implemented" [23]. At its simplest level, context may refer to the physical environment where health care takes place. However, Rycroft-Malone et al. concluded from their concept analysis that contexts conducive to research implementation included a range of less tangible process elements: "clearly defined boundaries; clarity about decision-making processes; clarity about patterns of power and authority; resources; information and feedback systems; active management of competing "force fields" ... and systems in place that enable dynamic processes of change and continuous development.' [23].

Theories underpinning organisational influence include those of learning organisations (with characteristics encompassing hierarchical structure, information systems, human resource practices, organisational culture and leadership [24]) and knowledge management (how organisational mechanisms affect knowledge uptake and use [25-27]). Successful implementation of an intervention to improve the management of post-stroke urinary incontinence is likely to be mediated not only by individual members of staff and availability of evidence-based guidance, but also by the complexity of the intervention as well as the interplay of patient, social and organisational factors [28,29]. Careful attention needs to be paid to the specific barriers to change in any given setting, identified through 'diagnostic analysis' [30] at levels that may include the individual, groups or teams, organisations and the wider healthcare system [31]. Strategies then need to be 'tailored' to overcome barriers identified [32].

We therefore plan to evaluate whether supported implementation, through targeted organisational development aimed at 'normalising' the intervention [33-35], is more effective than introduction of the intervention alone, as well as evaluating both in comparison to usual care.

### Aim

The trial aims to assess the feasibility of a full-scale cluster-randomised trial: to test the interventions for preliminary evidence of clinical effect and to provide information to enable estimates of the number of sites and patients that would need to be recruited for a full-scale cluster-randomised trial to evaluate effectiveness.

### Objectives

The trial objectives are to:

• assess feasibility in terms of rates of participant recruitment and retention

• assess fidelity to the intervention

• conduct a qualitative assessment of feasibility from the perspective of multiple stakeholders

• conduct a preliminary evaluation of supported implementation compared with implementation alone

• investigate patient-related factors affecting patient outcome

• investigate stroke service-level factors potentially affecting stroke service outcomes to estimate the amount of unexplained variability in outcomes between Trusts and between patients

• confirm the choice of primary and secondary outcome measures for a full-scale cluster-randomised trial to evaluate effectiveness

• develop and test data collection tools for an economic evaluation within a full-scale cluster-randomised trial

### Design

A three arm, parallel, open, exploratory, pragmatic, cluster-randomised controlled trial of a systematic voiding programme (including bladder training and pelvic floor muscle training for patients who are cognitively able and prompted voiding for patients with cognitive impairments), with or without supported implementation, for the management of urinary incontinence after stroke in secondary care.

## Methods

### Study setting

Twelve NHS stroke services in England and Wales. For the purpose of the trial, a stroke service will comprise both acute and rehabilitation stroke units. Stroke units will be defined according to the definition provided by the Royal College of Physicians of London for the National Sentinel Stroke Audit [7].

#### Inclusion criteria for stroke services

Stroke services with specialist acute and rehabilitation stroke services (either separate or combined units).

Access to appropriate Excess Treatment and Service Support costs.

#### Exclusion criteria for stroke services

Stroke service without specialist acute and rehabilitation stroke units (either separate or combined).

#### Inclusion criteria for patients

Patients aged 18 or over who have a diagnosis of stroke based on the WHO criteria [36] will be included in the study, with no upper age limit. Patients are required to have urinary incontinence (UI) as defined by the International Continence Society [37] as "involuntary loss of urine" and to have incontinence classified as stress UI, urge UI, mixed UI or 'functional' UI **OR **to be catheterised in the acute phase of the stroke; to be conscious (defined as either 'alert' or 'drowsy' on the 'Clinical Status on Admission' item of the European Stroke Database), and to be medically stable as judged by the clinical team.

Participants who had incontinence before the index stroke will be included. Given the expected age range of the population, there is likely to be a high prevalence of pre-stroke incontinence among potential participants. Furthermore, there is evidence to suggest patients with longstanding incontinence may benefit from a programme of behavioural interventions [9,11].

Participants who are catheterised will be recruited; if the catheter is removed, they will be assessed as per protocol and begin the systematic voiding programme if they are still incontinent. Participants who are continent after catheter removal and those discharged with a catheter still in situ will not be put onto the programme. Data from these participants will be analysed separately from participants who have taken part in the programme.

#### Exclusion criteria for patients

Patients who refuse consent.

Patients unable to consent for whom a consultee does not agree that the patient would wish to be included.

### Centre recruitment process

The trial has been adopted onto the Stroke Research Network (SRN) portfolio and was identified as open to new sites using SRN procedures. The research team also made contact with all stroke services in the North West of England and Wales and invited expressions of interest.

### Patient recruitment process

Patients who meet the inclusion criteria will be approached as early as possible following admission to the participating stroke unit. Patients who do not meet the inclusion criteria early post-stroke will be approached as soon as possible if they subsequently meet the inclusion criteria. Nursing staff will ask each eligible patient whether his/her name can be given to the research team. If the patient agrees, a member of the research team will visit the patient, explain the project, answer any questions they may have and provide an information leaflet. Patients will be given at least 24 hours to consider participation and will be visited by a member of the research team after this period; patients choosing to participate will sign the consent form at this stage.

For patients unable to consent for themselves, a person able to advise on the presumed wishes of the patient will be approached to act in the role of consultee. This is in line with the recommendations of the Mental Capacity Act [38] and in line with the expressed wishes of the study Patient, Public and Carer Involvement groups, who would like everyone who is eligible to have the opportunity to participate.

Recruitment rates in each cluster will be monitored on a monthly basis to identify both quantitative (i.e. numbers of participants recruited) and qualitative (i.e. patient characteristics) imbalance across clusters [39]. Should identified imbalance be indicative of likely selection bias (e.g. cluster with low proportion of stroke patients identified as eligible but high proportion of those eligible with severe incontinence), we will review the recruitment process and address any issues identified.

### Centre randomisation

#### Sequence generation

Stroke services will be placed into four strata based, in order of priority, on (i) whether they have separate or combined acute and rehabilitation units (ii) their average performance on the 'nine key indicators of stroke care' in the National Sentinel Stroke Audit Phase II (clinical audit) [7] (iii) the number of stroke patients admitted per year. Services will be randomly allocated to intervention (n = 4), intervention plus supported implementation (n = 4) and usual care (n = 4) groups by the Clinical Trials Unit at Newcastle University. After allocating hospitals to the strata, the randomisation schedule will be generated using block randomisation (block length of 3) to allocate one site to each arm within every stratum. The software package STATA (version 9) will be used.

##### Allocation concealment

Within each stratum, stroke services will not be informed as to their intervention allocation until ALL stroke services within that stratum are recruited to take part in the trial. However, services will then be aware of their allocation, as will staff identifying and recruiting trial participants from within that service.

### Interventions

#### Systematic voiding programme

The intervention will comprise algorithm-driven individualised systematic voiding programmes tailored to the physical and cognitive capabilities of each patient The algorithm specifies two routes: a combined package including bladder training and pelvic floor muscle training for those patients who are cognitively able, and prompted voiding for those with cognitive impairment. Bladder training will include three main components: 1) focused education for patients and carers (including information on the anatomy and physiology of the lower urinary tract, the rationale behind the programme and strategies to suppress the urge to void, for example distraction and relaxation [15,40]); 2) individualised voiding regimens designed to restore normal voiding patterns by progressively lengthening the time interval between voids, based on assessment of participants' normal voiding patterns and self-monitoring; 3) patient-held voiding diary, a cognitive intervention designed to promote self-awareness of voiding habits [41,42].

Pelvic floor muscle training is designed to strengthen types I and II muscle fibres in the pelvic floor. Patients will be instructed to perform 5 fast (3 seconds) and 10-20 sustained (10 seconds) contractions with 10 second relaxation periods between contractions twice a day.

For those patients with cognitive impairments, the programme will consist of elements traditionally classified as prompted voiding: participants will be approached according to individualised schedules (for example every two hours during waking hours), asked if they are wet or dry, and prompted to use the toilet [43]. Verbal praise will be offered for correct reporting of dryness/wetness and successful toileting. Participants with cognitive impairments will be given the opportunity to participate in the education and patient-held diary components of the intervention.

Participants not able to walk to the toilet will be assisted by nursing staff. Participants will be provided with written information about their systematic voiding programme to enable them to continue with it after discharge from hospital.

The intervention has been informed by the findings of an evidence synthesis on the barriers and enablers to successful implementation of conservative interventions for UI completed during the programme's Development Phase, in line with the MRC Framework for developing and evaluating complex interventions [44,45].

The systematic voiding programme will be incorporated into routine practice by all staff in day-to-day contact with patients. All nursing staff (including health care assistants, night staff and student nurses) will be provided with an education programme of both theory and practice. Training will be largely web-based to facilitate easy access and flexibility, but face-to-face sessions will also be offered to cover the practical aspects of intervention delivery and recording.

#### Systematic voiding programme plus supported implementation

Services randomised to the supported implementation arm of the trial will receive the systematic voiding programme together with supported implementation comprising: diagnostic analysis of context at the level of the organisation and the individual, identification of barriers (defined as "factors that impede the implementation of change in professional practice" [32]) and facilitators to the intervention, as well as targeted organisational development activities.

To support the process of implementing the systematic voiding programme, we will use a form of facilitation, a model that has been used successfully in secondary care settings [46,47]. The process of facilitation involves supporting and enabling people to change their practice [46-48]. Although approaches to facilitation vary, they are based on the principle that ownership is with the group [49]. The facilitator guides the group towards accomplishing a goal, helping members identify the obstacles that may impede progress and enabling members to identify strategies to overcome them [50]. While there will be a focus on goal attainment (defined as the normalisation of the systematic voiding programme [35]), our approach to facilitation will primarily focus on 'enabling' rather than 'doing for' others [47] with an emphasis on developing and empowering both individuals and teams. Experienced external facilitators will work with nominated internal facilitators in each site; further details of the supported implementation intervention are available on request from the lead author.

#### Usual care (control group)

Participants in this group will receive usual care provided by the stroke service. This may comprise: checking for urinary tract infection; checking for overflow incontinence (bladder scanners will be provided); containment using a variety of devices (for example absorbent products) with regular changes and some form of toileting schedule.

### Outcomes

#### Effectiveness

The primary effectiveness outcome will be incontinence (presence; absence). Measures will be taken at 6 weeks, 3 months and, for those recruited during the first three months, 12 months post stroke. The primary analysis will be of the 6-week data as conservative interventions for continence typically last six weeks [11,15], and an effect is most likely to be seen at this time point.

Secondary effectiveness outcomes will be: quality of life, frequency and severity of incontinence, urinary symptoms, activities of daily living (ADLs), and death; at 6 weeks, 3 months and, for those recruited during the first three months, 12 months post stroke.

#### Intervention fidelity

Intervention fidelity will be measured in terms of 1) percentage of occasions participants on the systematic voiding programme either self-initiated toileting or were prompted to toilet by ward staff within 30 minutes of the prescribed voiding interval and 2) accuracy of programme implementation, assessed by percentage of episodes where there was complete adherence to the elements of the protocol for the relevant conservative intervention (bladder training, prompted voiding and pelvic floor muscle training).

Data will be extracted from recording sheets specific to each route on the programme.

#### Ascertainment of outcomes

Presence/absence of incontinence will be measured by the International Consultation on Incontinence Questionnaire (ICIQ-UI Short Form) [51]. Absence of incontinence will be defined as the response 'never' to Question 3, 'How often do you leak urine?'; presence of incontinence will be defined as any other response to Question 3 (ranging from 'about once a week or less often' to 'all the time'). The ICIQ-UI Short Form has received a grading of A^new ^- highly recommended from the International Consultation on Incontinence Symptoms and Quality of Life Committee indicating published reports of acceptable reliability, validity and responsiveness in at least one study [52]. To our knowledge, the ICIQ-UI Short Form has not been used in the post-stroke population. We have conducted preliminary validation of the tool with six stroke survivors from our Patient, Public and Carer Involvement Group using the approach recommended by the ICIQ developers (Dr Nikki Cotterill, personal communication); all thought the tool was appropriate for use post-stroke and few problems were identified.

Quality of life will be measured using the Incontinence Quality of Life Instrument (I-QOL) [53,54] and the EuroQol (EQ-5D) [55]. Frequency and severity of incontinence will be ascertained using the Incontinence Severity Index [56]. Urinary symptoms will be measured using the Leicester Urinary Symptom questionnaire [57] and activities of daily living via the Barthel Index [58].

In addition, the following baseline information about the patient will be recorded following consent: date of birth (age to be calculated*); sex; ethnicity; date of admission; date of stroke onset; date baseline questionnaire completed; location when recruited into the study; consciousness level (defined as either 'alert' or 'drowsy' on the 'Clinical Status on Admission' item of the European Stroke Database); side of body affected by stroke; type of stroke; location of stroke (OCSP classification[59]); co-morbidities (Charlson Comorbidity Index[60]; day seven Barthel Index [58]; pre-stroke Modified Rankin Scale [61]*; pre-stroke living circumstances*; Leicester Urinary Symptom questionnaire [57]; type of UI (urge UI, mixed UI, 'functional' UI or unclear); cognitive ability (6 Item Cognitive Impairment Test [62]); fluid intake; bowel function; relevant clinical investigations (e.g. mid stream urine, bladder scan); medications; living circumstances; verbal sub-section of the Glasgow Coma Scale [63]*; ability to lift both arms off the bed*; ability to walk independently*; ICIQ-UI Short Form [51]; Incontinence Severity Index [56]; EuroQol (EQ-5D) [55].

The six factors highlighted with a * above from the Edinburgh stroke case mix adjuster [64] will be collected to enhance patient-level adjustment for prognosis.

#### Data collection

Baseline data will be collected on entry to the trial by the research nurse. Outcome data will be collected at six weeks, three months and (for participants recruited within the first three months of the trial) twelve months post-stroke via postal questionnaires (or hand-delivered questionnaires if participants are still in hospital at six weeks). All participants with aphasia will be offered a face-to-face interview with the research nurse to collect outcome data; this will be conducted using appropriate communication aids (e.g. pictorial cards with a 'thumbs up' picture indicating 'yes' and a 'thumbs down' card indicating 'no'). Specialist help will be available from Speech and Language Therapists and Speakeasy, a specialist aphasia charity based in Ramsbottom, Bury (http://www.buryspeakeasy.org.uk/), if this is needed.

Postal and telephone reminders will be used if questionnaires are not returned within two weeks. Where completion of postal questionnaires is not possible, participants (or carers if a proxy is needed) will be invited to complete assessments over the telephone. If neither postal or telephone completion is possible, a face-to-face assessment in the participant's home will be offered.

We will collect intervention fidelity data for all participants in the active intervention groups during three (for sites with a nine month intervention period) or four (for sites with a 12 month intervention period) randomly selected weeks during each three-month period in which the stroke service is recruiting participants into the trial.

#### Blinding

It is not possible for the health professionals or patients to be blinded to the intervention. However, data collectors will be blinded. The trial statistician will not be blinded during the analysis, however the statistical analysis plan will be finalised prior to any outcome data being available to the trial statistician.

### Qualitative evaluation of implementation

An integrated qualitative evaluation will be conducted in order to describe implementation and assist in explaining why the intervention and its components were or were not successful. Semi-structured interviews will be conducted with patients at discharge and will seek patients' experiences of their treatment for incontinence and their views of the effectiveness of treatment.

We will use maximum variance sampling [65] to generate a range of participants (around 15-20 from each trial arm, but with the numbers determined by data saturation) in terms of gender, age, ethnicity, type of incontinence and stroke severity. Patients will also be chosen to reflect those with a range of outcomes at discharge (defined in terms of the frequency of incontinent episodes).

Qualitative, semi-structured interviews will be performed with health professionals (n = 15-20 per intervention group, but with the numbers determined by data saturation) involved in the intervention to explore experiences of implementing the intervention and drivers and barriers to successful implementation. Qualitative interviews (n = 15-20) will also be held with health professionals in the 'usual care' trial arm to facilitate comparison in terms of continence management across trial arms.

### Sample Size

The sample size is chosen pragmatically, rather than on the basis of a formal power calculation; our aim is to balance practicalities and the need for reasonable precision in the estimation of effects to inform the sample size calculation for a full-scale trial to test effectiveness.

The use of four stroke services per arm (12 in total) will provide a satisfactory indication of likely effectiveness and help us address any feasibility issues relating to the delivery of the interventions. It will also provide some degree of confirmation regarding the size of the intra-class correlation coefficient and will enable us to perform a review of the number of sites (and the number of patients needed per site) for each arm of the trial for a full-scale trial. It will also provide some information on stroke service-level factors which may help explain the variability in outcome between stroke services in each arm, thus helping reduce the intra-class correlation coefficient and hence achieve efficiency in terms of the number of stroke services required for a full-scale trial.

Twelve stroke services will admit around 4800 patients per year, of whom we expect around 20% will meet the trial inclusion criteria and consent to participate. To achieve better balance in the number of participants recruited per Trust, we will recruit for 12 months in services which admit 300 or less patients per year and for 9 months in Trusts which admit more than 300 patients per year; we expect that this will enable us to recruit 780 patients across the 12 services.

### Data analysis

#### Patient baseline data

Baseline characteristics will be summarised using means (with standard deviations), or median (inter-quartile range) if quantitative (continuous or count) as appropriate, or frequency (percentage) if dichotomous or categorical. These will be summarised both within intervention groups and within clusters (within intervention groups). All outcome data will be summarised in a similar manner.

#### Outcomes

The primary analysis set will be defined as the intention-to-treat population. All stroke services randomised will be retained in the trial. Outcome data will be collected from all consented patients whenever possible, whatever their level of subsequent engagement with the allocated intervention programme. If there is more than 10% loss to follow-up overall or moderately differential loss to follow-up across stroke services, a secondary per-protocol analysis will also be considered. The decision as to whether a per-protocol analysis will be informative will be made by the trial statistician in conjunction with other members of the Trial Management Group; this decision will be made without reference to the recorded values of the outcome data.

To account for the cluster randomisation, we shall use mixed effects modelling for continuous, ordinal and dichotomous outcomes to compare the two groups on primary and secondary outcome data. Baseline measures of the outcomes variables (where appropriate), stroke classification, type of incontinence and the other prognostic patient-level information (from the Edinburgh case-mix adjuster) will be included as individual-level covariates in the models for outcome data.

Missing outcome data will be imputed according to the particular outcome. For the primary analysis, dichotomous and ordinal outcomes for those who withdraw, die or are otherwise lost to follow-up will be imputed using a worst-case scenario (e.g. for the primary outcome variable, all those for whom incontinence status is not recorded at 6-weeks post-stroke will be assumed to be incontinent). For continuous outcomes, the primary analysis will use a non-parametric multiple imputation approach [66].

The effect of stroke service-level factors will also be explored in the modelling; this will enable appropriate stratification to be used in the future larger scale trial, if desired, and to potentially reduce the size of the intra-stroke service correlation coefficient by reducing the unexplained component of the variability between stroke services. This analysis will be used to confirm the sample size necessary for the future trial and to provide estimates (including 95% confidence intervals) of the effectiveness of each intervention relative to usual care.

Various sensitivity analyses will be performed. These will assess: the robustness of assumptions inherent in the analyses (including the use of alternative analytical approaches given the small number of clusters and of alternative approaches to imputation of missing outcome data); the effect of excluding patients subsequently found **not **to have had a stroke from analyses and the impact on the estimate of effectiveness of including patients with pre-stroke incontinence.

#### Recruitment

Each month, proportions of stroke patients meeting the inclusion criteria for the trial and proportions of these patients actually recruited will be presented and compared between clusters and between intervention groups, both descriptively and inferentially using chi-square tests. Every three months we will also investigate descriptively whether there is an association between a cluster's reported eligibility rate and the percentage of recruited patients with mild incontinence using a range of appropriate techniques, including graphical representation and rank correlations.

#### Intervention fidelity data

For the intervention groups, fidelity measures will be summarised using mean (with standard deviation) or median (inter-quartile range), depending on the distributions of the percentage fidelity measures. Fidelity will be compared across intervention groups and across different conservative interventions (i.e. bladder training, prompted voiding and pelvic floor muscle training).

#### Qualitative data

Qualitative interviews will be audio taped, transcribed and subject to thematic analysis using FRAMEWORK [67] to identify emergent themes. FRAMEWORK method permits identification and cross-classification of variables from paper transcriptions. The analysis process consists of identifying key concepts and mapping their range and diversity, followed by a process of interpretation where patterns of association are investigated and possible reasons for these explored. The validity of the interpretation will be assessed through regular discussion by the project team.

### Economic evaluation

In this pilot trial, we will develop data collection tools to record resource use which will be used in a future, definitive trial. Tools will include: proformas for staff to record training and time spent on the intervention; a structured staff-observation schedule to allow independent assessment of time spent on the intervention; proforma to record resources (e.g. tests and investigations, length of stay) used in hospital (data will be obtained from the hospital information system); postal questionnaire about health and social service input, for self-completion by patients and carers. The postal questionnaires will be based on a design used previously by the applicants when querying input after discharge in a cohort of stroke patients.

Two measures will be used for the cost-utility analysis. The EuroQol (EQ-5D)[55], a generic quality of life measure, will be used to facilitate comparison with other studies and will allow us to estimate the benefit of the intervention in terms of quality adjusted life years (QALYs) gained. However, it has been suggested that the EQ-5D has a large ceiling effect and poor responsiveness in a non-stroke sample of women with urinary incontinence [68]. We will therefore also use a condition-specific measure, the Incontinence-Specific Quality of Life instrument (I-QOL, [52,53]), which has been shown to be the best continence-specific measure for use in clinical trials in terms of reliability, validity and responsiveness to change [67].

We will build on the cost-utility analysis by conducting an additional sensitivity analysis. We will review published economic evaluations that have estimated Quality Adjusted Life Years (QALYs) and extract the utility (quality-of-life) values they used. We will tabulate the basis for these figures in terms of the size of sample, which patients were questioned, what technique was used (EQ-5D or other), and how values were derived. We will use the data which seem to have the most validity in the sensitivity analysis.

### Ethical aspects

The trial has been approved by Bradford Research Ethics Committee (Reference number 10/H1302/60), which has a lead responsibility for studies with Mental Capacity issues, and by local Research and Development departments.

Informed consent to participate in the trial will be sought from participants themselves or from a consultee, as outlined above. All patients will be informed that participation is voluntary and that they are able to withdraw at any time. Urinary incontinence is a sensitive issue and we believe the approach taken with participants needs to reflect this. Our study has two dedicated Patient, Public and Carer Involvement groups, and these have provided advice on recruitment strategies as well as assisting in the development of patient documentation.

### Trial management and monitoring

The Management Group (comprising all programme applicants) meets every 3 months to discuss the day to day management and running of the programme. The Trial Steering Committee meets every 6 months with a remit including review of objectives and progress against plans and objectives and monitoring the quality of the research to help ensure it contributes to knowledge at a national and international level.

The Data Monitoring Committee (DMC) will be responsible for safeguarding the interests of trial participants, assessing the effect of the interventions during the trial, and for monitoring the overall conduct of the clinical trial. The DMC will be advisory to the Trial sponsor and the Trial Steering Group and will meet biannually.

## Abbreviations

ADLs: Activities of daily living; DMC: Data Monitoring Committee; EQ-5D: EuroQol quality of life measure; ICIQ-SF: International Consultation on Incontinence Questionnaire - Short Form; ICONS: Identifying Continence OptioNs after Stroke study I-QOL: Incontinence Quality of Life instrument; MRC: Medical Research Council; NHS: National Health Service; NIHR: National Institute for Health Research; OCSP: Oxford Community Stroke Project; PPC Groups: Patient, Public and Carer Involvement Groups; QALYS: Quality Adjusted Life Years; SRN: Stroke Research Network; UI: Urinary incontinence; UK: United Kingdom; WHO: World Health Organization

## Competing interests

The authors declare they have no competing interests.

## Authors' contributions

All authors were responsible for the study design. LT and CW were responsible for obtaining funding. LT is programme coordinator, responsible for the ongoing management of the trial, assisted by DF, the trial manager. CS developed the statistical analyses. LT wrote the initial draft manuscript. All authors have read and corrected the draft version and all authors contributed to and approved the final manuscript.

## Author details

^1^University of Central Lancashire, Preston, UK. ^2^Glasgow Caledonian University, Cowcaddens Road, Glasgow, UK. ^3^Edge Hill University, St Helens Road, Ormskirk, UK. ^4^Bangor University, Gwynedd, UK. ^5^Newcastle University, Medical School, Newcastle upon Tyne, UK.
